# Community-associated Methicillin-Resistant *Staphylococcus aureus* in Outpatients, United States, 1999–2006

**DOI:** 10.3201/eid1512.081341

**Published:** 2009-12

**Authors:** Eili Klein, David L. Smith, Ramanan Laxminarayan

**Affiliations:** Princeton University, Princeton, New Jersey, USA (E. Klein, R. Laxminarayan); University of Florida, Gainesville, Florida, USA (D.L. Smith); Resources for the Future, Washington, DC, USA (E. Klein, R. Laxminarayan)

**Keywords:** Methicillin-resistant Staphylococcus aureus, community-associated MRSA, hospital-associated MRSA, phenotypic susceptibility, outpatients, staphylococci, bacteria, CME, research

## Abstract

Community-associated methicillin-resistant *Staphylococcus aureus* (CA-MRSA) has become a major problem in US hospitals already dealing with high levels of hospital-associated MRSA (HA-MRSA). Using antimicrobial drug susceptibility data for 1999–2006 from The Surveillance Network, we characterized the relationship between outpatient and inpatient levels of CA-MRSA nationally. In outpatients, the frequency of CA-MRSA isolates has increased >7× during 1999–2006, which suggests that outpatients have become a major reservoir for CA-MRSA. However, contrary to results in other reports, although CA-MRSA increases are associated with decreases in the frequency of HA-MRSA in hospitals, the decreases are only modest. This finding suggests that instead of replacing HA-MRSA in the hospital, CA-MRSA is adding to the overall presence of MRSA already found within the hospital population.

## Abstract 2

These patients likely play a role in transmission of these organisms into hospitals.

## CME ACTIVITY

MedscapeCME is pleased to provide online continuing medical education (CME) for this journal article, allowing clinicians the opportunity to earn CME credit. This activity has been planned and implemented in accordance with the Essential Areas and policies of the Accreditation Council for Continuing Medical Education through the joint sponsorship of MedscapeCME and Emerging Infectious Diseases. MedscapeCME is accredited by the Accreditation Council for Continuing Medical Education (ACCME) to provide continuing medical education for physicians. MedscapeCME designates this educational activity for a maximum of 0.5 *AMA PRA Category 1 Credits*™. Physicians should only claim credit commensurate with the extent of their participation in the activity. All other clinicians completing this activity will be issued a certificate of participation. To participate in this journal CME activity: (1) review the learning objectives and author disclosures; (2) study the education content; (3) take the post-test and/or complete the evaluation at **http://www.medscape.com/cme/eid**; (4) view/print certificate.

## Learning Objectives

Upon completion of this activity, participants will be able to:

- Specify characteristics of community-acquired methicillin-resistant *Staphylococcus aureus* (MRSA) compared with hospital-acquired MRSA
- Recognize recent trends in MRSA among outpatients
- Identify anatomic sites most commonly associated with infection with MRSA resistant only to oxacillin
- Recognize recent trends in MRSA among inpatients
- Describe the most common treatment for rickettsial diseases.

## Editor

**Thomas Gryczan**, Technical Writer-Editor, *Emerging Infectious Diseases. Disclosure: Thomas Gryczan has disclosed no relevant financial relationships.*

## CME AUTHOR

**Charles P. Vega, MD**, Associate Professor; Residency Director, Department of Family Medicine, University of California, Irvine. *Disclosure: Charles P. Vega, MD, has disclosed no relevant financial relationships.*

## AUTHORS

Disclosures: **Eili Klein, MA; David L. Smith, PhD;** and **Ramanan Laxminarayan, PhD, MPH,** have disclosed no relevant financial relationships.

## Earning CME Credit

To obtain credit, you should first read the journal article. After reading the article, you should be able to answer the following, related, multiple-choice questions. To complete the questions and earn continuing medical education (CME) credit, please go to **http://www.medscape.com/cme/eid**. Credit cannot be obtained for tests completed on paper, although you may use the worksheet below to keep a record of your answers. You must be a registered user on Medscape.com. If you are not registered on Medscape.com, please click on the New Users: Free Registration link on the left hand side of the website to register. Only one answer is correct for each question. Once you successfully answer all post-test questions you will be able to view and/or print your certificate. For questions regarding the content of this activity, contact the accredited provider, CME@medscape.net. For technical assistance, contact CME@webmd.net. American Medical Association’s Physician’s Recognition Award (AMA PRA) credits are accepted in the US as evidence of participation in CME activities. For further information on this award, please refer to http://www.ama-assn.org/ama/pub/category/2922.html. The AMA has determined that physicians not licensed in the US who participate in this CME activity are eligible for *AMA PRA Category 1 Credits*™. Through agreements that the AMA has made with agencies in some countries, AMA PRA credit is acceptable as evidence of participation in CME activities. If you are not licensed in the US and want to obtain an AMA PRA CME credit, please complete the questions online, print the certificate and present it to your national medical association.

### Article Title: Community-associated Methicillin-Resistant *Staphylococcus aureus* in Outpatients, United States, 1999–2006

## CME Questions

1. Which of the following characteristics helps to differentiate community-associated methicillin-resistant *Staphylococcus aureus* (CA-MRSA) from hospital-associated (HA)–MRSA?
  A. Resistance to fluoroquinolones
  B. Resistance to a higher number of antibiotics
  C. Resistance to vancomycin
  D. Resistance to beta-lactam and erythromycin only
2. Which of the following trends were noted in the epidemiology of *outpatient* MRSA in the current study?
  A. The presence of MRSA was stable over the study period
  B. The number of MRSA isolates resistant to at least 1 other drug increased significantly
  C. *S aureus* infections that were MRSA nearly doubled
  D. HA-MRSA accounted for the majority of change in the prevalence of MRSA among outpatients
3. The number of *S aureus* isolates resistant only to oxacillin increased most significantly from which anatomic site?
  A. Lung
  B. Blood
  C. Skin and soft tissue
  D. Genitourinary tract
4. Which of the following statements about the epidemiology of MRSA among *inpatients* is most accurate?
  A. The proportion of *S aureus* infections that were MRSA increased by 25%
  B. The prevalence of *S aureus* isolates resistant only to oxacillin decreased
  C. The prevalence of HA-MRSA isolates fell sharply as CA-MRSA increased
  D. There was a significant increase in lung infections with multiple-drug resistant MRSA

### Activity Evaluation

**Table:** 

**1. The activity supported the learning objectives.**				
Strongly Disagree				Strongly Agree
1	2	3	4	5
**2. The material was organized clearly for learning to occur.**				
Strongly Disagree				Strongly Agree
1	2	3	4	5
**3. The content learned from this activity will impact my practice.**				
Strongly Disagree				Strongly Agree
1	2	3	4	5
**4. The activity was presented objectively and free of commercial bias.**				
Strongly Disagree				Strongly Agree
1	2	3	4	5

## Community-associated Methicillin-Resistant *Staphylococcus aureus* in Outpatients, United States, 1999–2006

The past decade has seen a large increase in infections with hospital-associated methicillin-resistant *Staphylococcus aureus* (HA-MRSA) (*1*). MRSA is one of the most common causes of nosocomial infections, especially invasive bacterial infections (*2*), and is now endemic and even epidemic to many US hospitals, long-term care facilities (*3*), and communities (*4*–*6*). Although community-associated MRSA (CA-MRSA) strains have been recognized as a leading cause of skin and soft tissue infections (*1*,*6*), especially in patients with no established healthcare risk factors (*7*,*8*), they also cause severe invasive infections (*9*,*10*). Recent reports based on genotypic evidence have suggested that CA-MRSA is likely spreading within hospitals as well, blurring the line between CA-MRSA and HA-MRSA infections (*11*).

Molecular typing studies have identified 2 MRSA clones, USA300 and USA400, as the primary types that cause CA-MRSA infections (*12*). Evidence suggests that emergence of these strains was independent of hospital strains (*13*). Thus, understanding the role of outpatients, who are among the likely carriers of CA-MRSA into a hospital, is useful for understanding the changing epidemiology of MRSA in hospitals. Outpatients, who outnumber inpatients by ≈3:1, may play a major role in the spread of CA-MRSA strains from the community to the hospital through their interaction with hospital staff or use of similar hospital resources, such as surgical rooms. However, limited information hinders understanding of long-term trends in CA-MRSA in outpatients in the context of changing epidemiology of inpatients. This lack of information hinders the ability to evaluate infection control methods in the face of a possible emerging epidemic of nosocomial infections caused by CA-MRSA.

Knowledge of trends in antimicrobial drug resistance rates for emerging pathogens are useful to clinicians to ensure high-quality care, which is essential for antimicrobial drug therapy, in which different drugs can have different costs and effectiveness. These trends can also help hospital administrators and policy makers make infection control investments to address the role that large influxes of outpatients with CA-MRSA infections may play with regard to overall MRSA infection rates in the hospital.

## Methods

To analyze trends in frequency of CA-MRSA and HA-MRSA, we studied changes in the proportion of isolates of each type that were found in inpatient and outpatient settings from a nationally representative sample of US hospitals during 1999–2006. Although genotypic analysis is the most reliable way of identifying MRSA strains, historical genotypic data on isolates are not available at the national level. An alternative approach is to ascertain strain type by using phenotypic susceptibility profiles. *S*. *aureus* susceptibility profiles are determined by the staphylococcal cassette chromosome (SCC) types on which the methicillin resistance gene, *mec*A, is carried. Because CA-MRSA and HA-MRSA strains typically have different SCC*mec* types, rules have been developed for determining the likely genetic makeup of an isolate on the basis of susceptibility results (*11*,*14*–*16*).

Phenotypic susceptibility results were obtained from The Surveillance Network (TSN) Database-USA (Focus Diagnostics, Herndon, VA, USA). TSN is an electronic repository of antimicrobial drug susceptibility data from a national network of >300 microbiology laboratories in the United States. Participating laboratories are geographically dispersed and make up a nationally representative sample based on patient population and number of beds. Patient isolates are tested on site as part of routine diagnostic testing for susceptibility to different antimicrobial agents by using standards established by the Clinical and Laboratory Standards Institute (*17*) and approved by the US Food and Drug Administration. Results are then filtered to remove repeat isolates and identify microbiologically atypical results for confirmation or verification before being included in the TSN database. Data from the database have been used extensively to evaluate antimicrobial drug resistance patterns and trends (*1*,*18*–*22*).

Genotypic analysis of phenotypically defined strains has found that in general, isolates of the USA300 strain, the one most commonly associated with CA-MRSA infections, are resistant to fewer antimicrobial drugs (*14*–*16*). Naimi et al. (*15*) tested genetically determined CA-MRSA isolates against several antimicrobial drugs and found that they were typically susceptible to ciprofloxacin (79%) and clindamycin (83%). Similarly, King et al. (*14*) found that 88% of CA-MRSA strains were resistant only to a β-lactam and erythromycin or a β-lactam only. Popovich et al. (*16*) also found that susceptibility to a fluoroquinolone had a 90% positive predictive value for predicting a community-associated strain. Additionally, the number of antimicrobial drugs to which an isolate was susceptible was a reliable predictor of the genotype (*16*).

We analyzed *S*. *aureus* isolates that were tested for susceptibility to oxacillin (a proxy for all β-lactam antimicrobial drugs). Isolates classified as resistant according to Clinical and Laboratory Standards Institute breakpoint criteria were considered MRSA (<0.01% had intermediate resistance and were classified as susceptible). MRSA isolates, regardless of source (outpatient or inpatient), that were tested against ciprofloxacin or clindamycin, and >3 other drugs and found to be resistant only to oxacillin were classified as CA-MRSA strains. Isolates resistant to oxacillin and >1 other drug were assumed to be HA-MRSA strains. Other drugs tested were gentamicin, tetracycline, sulfamethoxazole/trimethoprim, and vancomycin.

Using this framework, we determined that the mean number of outpatient isolates analyzed annually was >50,000. Isolates were stratified on the basis of source (blood, lungs, skin, and other organs). Confidence intervals (CIs) for TSN data were calculated by using the Wilson score method incorporating continuity correction as detailed by Newcombe (*23*). Statistical analysis was performed by using Stata version 10 software (StataCorp LP, College Station, TX, USA).

## Results

Susceptibility to clindamycin, ciprofloxacin, gentamicin, tetracycline, sulfamethoxazole/trimethoprim, and vancomycin was used to infer genotypes of MRSA isolates during 1999–2006. During this period, there was a statistically significant reduction (p<0.001) in the number of MRSA isolates in outpatient areas resistant to ciprofloxacin (84% to 56%), clindamycin (67% to 30%), gentamicin (30% to 3%), and sulfamethoxazole/trimethoprim (16% to 2%). Our phenotypic rule, which was based on susceptibility to all drugs, found qualitatively similar results, with the number of MRSA isolates resistant to >1 other drug decreasing from 87% to 46% during the period (Figure).

**Figure:**
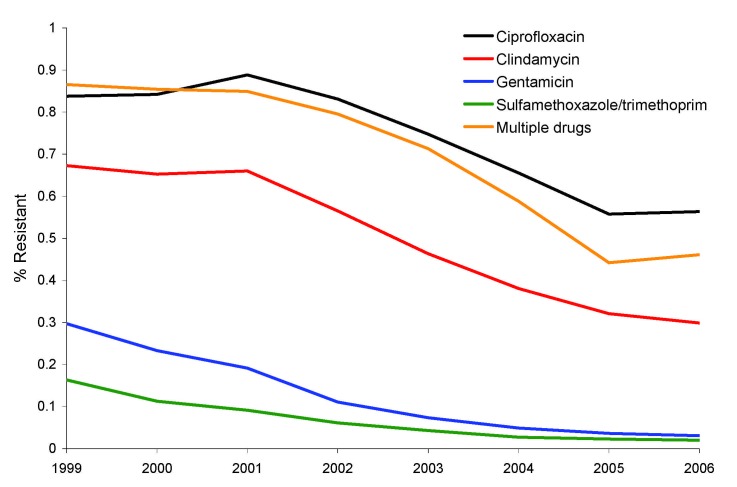
Resistance of methicillin-resistant *Staphylococcus aureus* isolates to clindamycin, ciprofloxacin, gentamicin, and sulfamethoxazole/trimethoprim in outpatient areas of hospitals, United States, 1999–2006. Multiple drugs indicates isolates that were tested against ciprofloxacin or clindamycin and >3 other drugs and found to resistant only to oxacillin. The p values were calculated by using the χ^2^ test. Differences in all comparisons were significant (p<0.001).

For outpatient data, the proportion of all *S*. *aureus* infections that were MRSA infections nearly doubled, from 26.8% (95% CI 26.3%–27.3%) to 52.4% (95% CI 52.0%–52.9%), over the study period. This increase was caused almost entirely by increases in isolates resistant only to oxacillin, which increased >7× from 3.6% (95% CI 3.5%–3.7%) to 28.2% (95% CI 28.0%–28.5%). The proportion of isolates resistant only to oxacillin increased for skin and soft tissue infections. However, increases were also observed in invasive blood and lung infections and other infections. Isolates resistant to >1 other drug increased ≈5% during 1999–2001 from 23.2% (95% CI 23.0%–23.5%) to 28.2% (95% CI 28.0%–28.5%) before reaching a plateau. In 2005, the proportion of isolates resistant to oxacillin and 1 other drug then decreased back to almost the same percentage it started at. This pattern was driven by overall increases at all infection sites during 1999–2001 and later decreases at all collection sites except skin infections (Table).

**Table:** Frequency of MRSA in hospitals, by unit, United States, 1999–2006*

Unit	% Patients (95% confidence interval)							
	1999	2000	2001	2002	2003	2004	2005	2006†
Outpatient								
All MRSA	26.8 (26.3–27.3)	29.4 (29.0–29.9)	33.4 (33.0–33.9)	35.7 (35.3–36.2)	40.7 (40.2–41.2)	47.7 (47.3–48.1)	52.7 (52.3–53.1)	52.4 (52.0–52.9)
HA-MRSA	23.2 (23.0–23.5)	25.1 (24.9–25.4)	28.2 (28.0–28.5)	28.4 (28.2–28.7)	29.3 (29.0–29.5)	28.4 (28.2–28.7)	24.1 (23.8–24.3)	24.2 (24.0–24.5)
Blood	2.7	2.8	4.0	3.7	3.1	2.8	2.1	1.9
Lungs	4.7	5.3	6.5	5.5	5.4	4.2	3.5	2.9
Skin	9.3	10.6	10.1	11.6	13.8	15.5	14.2	15.5
Other source	6.4	6.5	7.6	7.7	7.0	6.0	4.2	4.0
CA-MRSA	3.6 (3.5–3.7)	4.3 (4.2–4.4)	5.2 (5.1–5.4)	7.3 (7.1–7.5)	11.4 (11.3–11.6)	19.3 (19.1–19.5)	28.7 (28.4–28.9)	28.2 (28.0–28.5)
Blood	0.3	0.3	0.6	0.7	0.8	0.8	0.9	0.8
Lungs	0.4	0.5	0.9	0.9	0.7	0.6	0.8	0.7
Skin	2.3	3.0	2.9	4.8	9.0	16.6	25.3	25.4
Other source	0.5	0.5	0.8	0.9	1.0	1.3	1.6	1.4
Inpatient								
All MRSA	46.7 (46.2–47.2)	47.6 (47.2–48.1)	50.0 (49.6–50.4)	52.2 (51.8–52.6)	54.9 (54.6–55.3)	58.3 (57.9–58.6)	59.5 (59.2–59.9)	58.5 (58.0–58.9)
HA-MRSA	43.4 (43.0–43.9)	43.2 (42.8–43.6)	44.1 (43.7–44.5)	43.9 (43.5–44.3)	44.1 (43.7–44.5)	41.9 (41.6–42.3)	38.5 (38.2–38.9)	38.7 (38.3–39.1)
Blood	7.1	7.2	7.5	7.5	7.5	7.0	6.2	6.3
Lungs	21.4	19.9	19.2	18.5	17.6	16.5	14.7	14.5
Skin	9.3	10.5	11.4	12.0	12.9	13.1	13.0	13.1
Other source	5.6	5.6	6.0	5.9	6.1	5.3	4.5	4.7
CA-MRSA	3.3 (3.1–3.4)	4.5 (4.3–4.6)	5.8 (5.6–6.0)	8.4 (8.2–8.6)	10.9 (10.6–11.1)	16.3 (16.1–16.6)	21.0 (20.7–21.3)	19.8 (19.4–20.1)
Blood	0.6	0.7	1.0	1.2	1.2	1.7	2.1	2.0
Lungs	1.2	1.6	2.1	2.3	2.1	2.6	3.3	3.8
Skin	1.1	1.7	2.1	4.0	6.6	10.9	14.4	12.8
Other source	0.4	0.5	0.7	0.9	1.0	1.2	1.3	1.2

*MRSA, methicillin-resistant *Staphylococcus aureus*; HA-MRSA, hospital-associated MRSA; CA-MRSA, community-associated MRSA. †Data through October only. Data for blood, lungs, skin, and other source refer to the percentage of each source tested that was estimated to be CA-MRSA or HA-MRSA.

Among inpatients, the proportion of *S*. *aureus* isolates that were MRSA increased 25% from 46.7% (95% CI 46.2%–47.2%) to 58.5% (95% CI 58.0%–58.9%). Again, the increase was driven primarily by increases in the rate of isolates resistant only to oxacillin, which increased >7× from 3.3% (95% CI 3.1%–3.4%) to 19.8% (95% CI 19.4%–20.1%). Similar to outpatient data, the frequency of skin and soft tissue infections increased for isolates resistant only to oxacillin, although increases in blood, lung, and other infections were also observed. For isolates resistant to >1 other drug, a slightly different pattern was observed than for the pattern of outpatient isolate resistance. Instead of a large increase, the proportion of MRSA isolates resistant to >1 drug remained the same (≈43%–44%) until 2003 before decreasing >5% from 44.1% (95% CI 43.7%–44.5%) to 38.5% (95% CI 38.2%–38.9%) during 2003–2005. This decrease was largely caused by reductions in lung infections, although decreases were also seen in blood and other infections. Also different was the increase in MRSA skin isolates resistant to multiple drugs. There was an increase from 1999, but the increase was less (only 3%–4%) and appeared to plateau at ≈12%–13%.

## Discussion

We found during 1999–2006 that the percentage of *S*. *aureus* infections resistant to methicillin increased >90%, or ≈10% a year, in outpatients admitted to US hospitals. This increase was caused almost entirely by CA-MRSA strains, which increased >33% annually. Increases in the proportion of HA-MRSA isolates among outpatients were more variable, increasing ≈10% per year during 1999–2001 before the increase slowed; the proportion then decreased over the second half of the study period. This reduction in the growth of HA-MRSA isolates corresponds to a steep increase in the frequency of CA-MRSA skin and soft tissue infections among outpatients over an extremely short period, mostly during 2003–2005.

The frequency of CA-MRSA among inpatients increased nearly in conjunction with outpatient rates, overall and at each infection site. However, increases in blood and lung infections increased more among inpatients than in outpatients, which likely reflected the more severe status and increased likelihood of open wounds in inpatients. During this same period, rates of HA-MRSA decreased only ≈10%. Most of this decrease occurred during 2003–2005 and was mainly the result of a decrease in the frequency of HA-MRSA lung infections. This decrease was more likely the result of changes in empirical antimicrobial drug therapy for ventilator-associated pneumonia (*24*) than a consequence of any changes in the epidemiology of MRSA.

Despite increases in the proportion of CA-MRSA strains among inpatients, the continuing high level of HA-MRSA suggests that in contrast to reports from local institutions (*11*), CA-MRSA strains are adding to the problem of MRSA rather than replacing HA-MRSA strains. The fact that the frequency of HA-MRSA has decreased implies that some crowding out of HA-MRSA strains within the hospital may be occurring. However, lack of a decrease suggests that within the hospital, HA-MRSA strains may be more fit, and thus CA-MRSA strains are unable to replace them fully. The result is a coexistence of both strains in the hospital and maintenance of CA-MRSA because of the large influx of colonized and infected patients.

This finding is consistent with the biology of the 2 strains, which suggests differential fitness on the basis of the size of SCC*mec*. In CA-MRSA strains, the predominant SCC*mec* elements are types IV and V, which are smaller than the SCC*mec* types typically found in HA-MRSA strains. These smaller genetic elements may increase the fitness of CA-MRSA strains outside hospital-related antimicrobial drug pressures, presumably by increasing mobility and growth potential (*25*). However, their increased susceptibility to antibacterial agents in the hospital leaves them at a fitness disadvantage. The result is that although the community has effectively become a reservoir for the CA-MRSA strains that are continually introduced into the hospital population without genetic changes, they are unlikely to replace HA-MRSA strains in the hospital.

The large proportion of infections caused by CA-MRSA strains in hospitals with high frequencies of HA-MRSA has implications for drug-prescribing patterns within hospitals. Because CA-MRSA strains are generally susceptible to more antimicrobial drugs, persons with these infections may be able to be treated with less expensive antimicrobial drugs with fewer adverse outcomes. Moreover, appropriate therapy can reduce the likelihood of emergence of other resistant pathogens, such as vancomycin-resistant enterococci. Initial empiric therapy of infections with the suspected etiology of CA-MRSA must be tailored to antimicrobial drug susceptibility patterns within the local community and be based on efficacy studies that suggest specific effectiveness targets.

Kaplan suggested that empiric therapy should be modified if >10%–15% of CA-MRSA isolates become resistant to a specific empiric therapy (*26*). Conversely, it may be appropriate to reintroduce a specific agent when susceptibility levels increase above a threshold. However, cycling strategies may not always be optimal (*27*), and no efficacy studies have been conducted to establish this target. In addition, we urge caution in applying national results to the CA-MRSA antibiogram of a specific area. Although results showed an overall trend at the national level, specific results at individual testing centers tended to be more variable. Moreover, local health officials and hospitals should coordinate their efforts to identify susceptibility patterns at the community level, rather than at the hospital level, to optimize the gains from investments in infection control (*28*).

The results of our study should be interpreted with caution because TSN provides information concerning only the site of isolate collection and not the infection. In addition, TSN only provides information on the collection location (i.e., outpatient or inpatient) and not case histories. Thus, some isolates may be difficult to classify in situations such as when an isolate was collected in the emergency department and then the patient was admitted or the patient was discharged and then returned as an outpatient. However, the effect of these situations is likely to be small because most isolates are from patients who can be classified as inpatients or outpatients.

A further limitation of the study is that although CA-MRSA isolate drug susceptibility patterns are technically genetically determined, the data enabled only phenotypic classification of isolates. In addition, as with any large time-series database, changes in surveillance or bias in the types of infections cultured over time, such as more severe or unusual infections, could alter the results. These findings suggest that more complicated bacteriology could alter the results. However, no general trend in the number of isolates collected was seen at individual testing centers, and resistance results from the TSN database were comparable to results of other national studies (*1*). Furthermore, the striking increases over the study period suggest that the trends are likely robust to any bias.

In summary, we examined the frequency of CA-MRSA and HA-MRSA in inpatient and outpatient settings. Our results indicate that outpatients may be a major reservoir of CA-MRSA, which will continue to enter hospitals, exacerbating the problem of MRSA. However, although CA-MRSA isolates have undoubtedly spread within hospitals and are likely to continue to do so, without changes in the fitness of different strains, CA-MRSA strains are unlikely to displace HA-MRSA strains within the hospital.

Our findings have implications for local and national policies aimed at containing and preventing MRSA. More rapid diagnostic methods are urgently needed to better aid physicians in determining appropriate empiric therapy. Strategies for prevention of infection and treatment of patients with CA-MRSA within healthcare settings should be coordinated primarily at the local level in accordance with local susceptibility profiles. Lastly, infection control policies should take into account the role that outpatients likely play in the spread of MRSA and promote interventions that could prevent spread of MRSA from outpatient areas to inpatient areas.
